# Overexpression, Purification, and Biochemical Characterization of the vanC2 d-Ala-d-Ser Ligase from *Enterococcus casseliflavus* SSK and Its Inhibition by an Oxadiazole Derivative

**DOI:** 10.1021/acsomega.5c00599

**Published:** 2025-04-03

**Authors:** Sneha B. Paymal, Sagar S. Barale, Shirishkumar V. Supanekar, Kailas D. Sonawane, Kiran D. Pawar

**Affiliations:** a Department of Microbiology, Shivaji University, Vidyanagar, Kolhapur, Maharashtra 416004, India; b Rayat Institute of Research and Development (RIRD), Satara, Maharashtra 415001, India; c Department of Microbiology, School of Life Sciences, Central University of Rajasthan, Ajmer, Rajasthan 305817, India; d Department of Biochemistry, Shivaji University, Vidyanagar, Kolhapur, Maharashtra 416004, India; e School of Nanoscience and Biotechnology, Shivaji University, Vidyanagar, Kolhapur, Maharashtra 416004, India

## Abstract

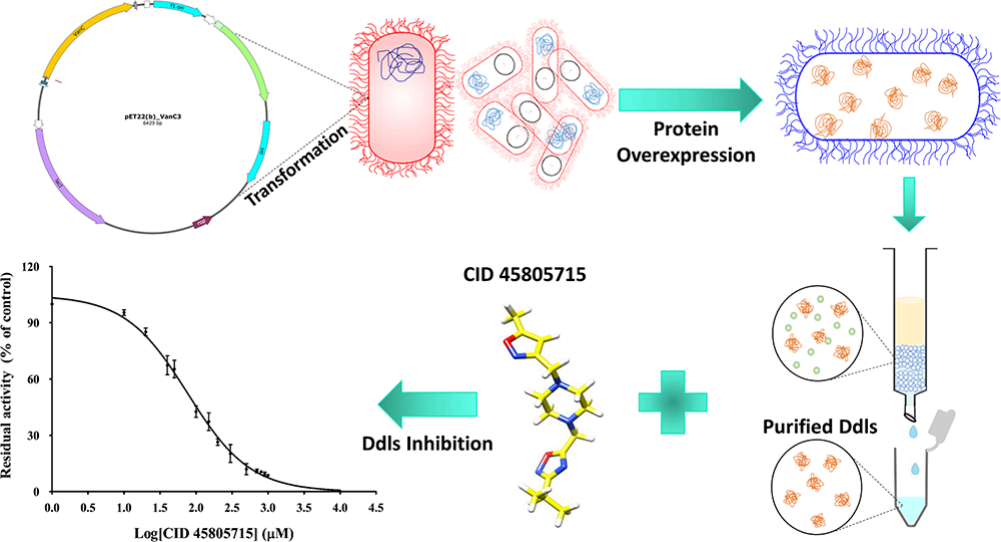

The bacterial cell wall and enzymes involved in peptidoglycan biosynthesis are prime targets for the discovery of novel antibacterial agents. Among these enzymes, d-alanine-d-alanine ligases (Ddl) are particularly significant due to their utilization of specific substrates (d-amino acids) essential for bacterial viability. Isozymes of Ddl that utilize alternative substrates such as d-lactate or d-serine are found in vancomycin-resistant Gram-positive bacteria, initially identified in Enterococcus species, and now represent a growing concern in clinical settings. In this study, we isolated and identified vancomycin-resistant *Enterococcus casseliflavus* (*E. casseliflavus*) strain SSK and used it for amplification, cloning, and purification of the vanC2 type of d-alanine-d-serine ligase (EcfDdls). Investigations of substrate specificity and enzyme kinetics provided insights into the enzyme’s mechanistic action. Evaluation of the inhibitory potential of the previously virtually screened oxadiazole derivative 1-[(5-methyl-1,2-oxazol-3-yl)methyl]-4-{[3-(propan-2-yl)-1,2,4-oxadiazol-5-yl]methyl}piperazine (CID 45805715) was carried out using an inorganic phosphate detection assay, which demonstrated complete enzymatic inhibition of purified EcfDdls. When tested, CID 45805715 significantly inhibited activity of Ddl, with an IC_50_ of 76.7 μM, compared to 313 μM for the reference compound DCS. Moreover, this compound also exhibited antimicrobial activity against vancomycin-resistant *E. casseliflavus* strain SSK. Thus, these findings provide valuable insights into the activity and inhibition of vanC2 EcfDdls, offering a promising avenue for addressing vancomycin resistance in enterococci, particularly in nosocomial infections affecting immunocompromised patients.

## 1 Introduction

Antibiotic resistance is a severe worldwide public health issue.^1^ It is associated with currently used various antibiotics rather than being limited to only one type. This potential to develop multidrug resistance leads to the emergence of organisms insensitive to existing drugs.^1^ Over 700,000 deaths worldwide are attributed to antibiotic-resistant bacterial infections each year, with a predicted increase to over 10 million by 2050. Various mechanisms contribute to the development of antibiotic resistance in bacteria.^2,3^ Vancomycin, a glycopeptide antibiotic, was once considered a “last resort drug” for controlling antibiotic-resistant Gram-positive bacteria. Since its discovery in 1952 by E.C. Kornfield, vancomycin has been successfully used for over 30 years to treat severe infections of methicillin-resistant *Staphylococcus aureus*.^4^ The first clinical isolates of vancomycin-resistant enterococci, *Enterococcus faecium* (*E. faecium*) and *Enterococcus faecalis* (*E. faecalis*), were identified in Great Britain in 1988, followed by other European countries and the United States.^5^ Vancomycin-resistant Enterococcus spp. (VRE) is a significant antibiotic-resistant concern, accounting for about 54,500 drug-resistant enterococcal infections and nearly 5400 deaths in the United States in 2017.^6^ According to the Centers for Disease Control and Prevention (CDC, U.S.), vancomycin resistance is present in about 30% of healthcare-associated enterococcal infections in the U.S.^6^ In 2017, 39.3–46.8% of clinical isolates in Australia were found to be vancomycin-resistant, making it one of the most prevalent in the world.^7^ The most recent annual epidemiological report by the European Centre for Disease Prevention and Control (ECDC) stated that the population-weighted mean fraction of vancomycin-resistant *E. faecium* isolates increased from 16.2% in 2018 to 17.6% in 2022.^8^ Studies in Germany showed that hospital VRE prevalence increased to over 20%, but studies in The Netherlands showed resistance rates of <1%. This discrepancy could be attributed probably to variations in antibiotic usage and infection prevention and control measures.^9^ Furthermore, *E. faecium* isolated from a number of other European countries such as Lithuania, Cyprus, Romania, Greece, Hungary, Croatia, Slovakia, and Ireland exhibited higher levels of vancomycin resistance in the range of 27.6–66.4%.^10,11^

Vancomycin resistance primarily results from isotypes of the d-Ala-d-Ala ligase enzyme, i.e., d-Ala-d-Ser ligase and d-Ala-d-Lac ligase altering the terminal dipeptide sequence to d-Ala-d-Ser (dipeptide) or d-Ala-d-Lac (depsipeptide), respectively, rather than d-Ala-d-Ala (dipeptide) during peptidoglycan synthesis.^12,13^ This alteration of d-alanine to d-serine or d-lactate results in a 6–1000-fold drop in affinity for vancomycin at the uncross-linked pentapeptide terminal.^14,15^ To date, eight distinct acquired vancomycin resistance types (vanA, vanB, vanD, vanE, vanG, vanL, vanM, and vanN) have been reported in Enterococci spp., with only the vanC type having intrinsic vancomycin resistance. In vanA, B, D, and M types, the gene encodes a d-Ala-d-Lac ligase that catalyzes the synthesis of a d-Ala-d-Lac depsipeptide,^5,16^ while in vanC-, E-, G-, L-, and N-type strains, the gene encodes a d-Ala-d-Ser ligase that catalyzes the synthesis of a d-Ala-d-Ser dipeptide.^17−21^ There is evidence that only motile enterococci, such as *Enterococcus gallinarum* (*E. gallinarum*), *Enterococcus casseliflavus* (*E. casseliflavus*), and *Enterococcus flavescens* (*E. flavescens*), exhibit the vanC phenotype.^22−24^ vanC1, vanC2, and vanC3 are three subtypes of the *vanC* gene, found *inE. gallinarum*, *E. casseliflavus*, and *E. flavescens*, respectively.^25,26^ Some Enterococcus species, like *E. faecalis* and *E. faecium*, are increasingly common causes of nosocomial infections, while others like *E. casseliflavus* have also been shown to be pathogenic to humans.^27^ In 1984, *E. casseliflavus* was given species status; it is a motile microorganism that differs from *E. gallinarum* by producing a yellow pigment.^27,28^*E. casseliflavus* is a rare pathogen found in clinical samples; however, it is an opportunistic pathogen that targets immunocompromised or chronically ill individuals and is acquired nosocomially.

The most prevalent type of *E. casseliflavus* infection is bacteremia.^29^ Retrospective cohort studies showed that solid tumors were a prevalent consequence in 40.12–48.8% of patients with bacteremia caused by *E. casseliflavus*.^29,30^ The most common complications in bacteremia cases caused by *E. casseliflavus* and *E. gallinarum* were malignancy (70%) and diabetes mellitus (20%).^31^ These findings suggest that immunocompromised hosts are at a higher risk of developing *E. casseliflavus* bacteremia. According to case reports, people between the ages of 60 and 80 are most susceptible to this bacteremia; however, there have also been reports of septicemia in neonatal cases.^32^ Several scientists, notably Ratanasuwan et al.,^33^ Reid et al.,^31^ and Choi et al.,^29^ have reported that vancomycin medication is contraindicated for VanC VRE infection, even for strains that were sensitive *in vitro*. In these instances, breakthrough bacteremia is associated with vancomycin therapy. Therefore, it is necessary to search for potential drugs against vancomycin-resistant Enterococci species to treat such infections. Since an alternative DD-ligase is the prime cause of resistance in vancomycin-resistant bacteria, pinpointing a DD-ligase inhibitor would be a key strategy in dealing with vancomycin resistance. To date, four main types of Ddl inhibitors have been reported: analogs of the substrate, analogs of the product, analogs of the transition state, and compounds obtained with the assistance of virtual screening or high-throughput screening (HTS) of chemical libraries in the drug designing process.^34^ The FDA has approved only d-cycloserine (DCS), the substrate analogue of d-Ala, as an inhibitor of Ddl for use as a second-line therapeutic agent against MDR (multidrug-resistant) *Mycobacterium tuberculosis* (*Mycobacterium tuberculosis*). However, the neurotoxic side effects of DCS have restricted its use in medicine.^35,36^

In this study, we isolated and characterized the vancomycin-resistant *Enterococcus casseliflavus* and studied its drug-resistant mechanisms. Through this investigation, we found the *vanC2* gene as the causative agent behind this resistance phenomenon. Further, we amplified, cloned, and overexpressed the *vanC2* gene in the pET22b(+) vector and purified the vanC2 d-Ala-d-Ser ligase. In addition, the enzyme kinetics and substrate specificity analyses revealed a distinct affinity of EcfDdls for d-Ala-d-Ser dipeptide synthesis. Furthermore, we evaluated the inhibitory potential of a previously virtually screened and identified candidate, 1-[(5-methyl-1,2-oxazol-3-yl)methyl]-4-{[3-(propan-2-yl)-1,2,4-oxadiazol-5-yl]methyl}piperazine (CID 45805715), alongside DCS, through determination of IC_50_ and MIC values.

Thus, the study reports identification, purification, and characterization of EcfDdls while simultaneously revealing the inhibitory and antibacterial efficacy of an oxadiazole derivative. This study not only helps to understand the mechanism of vancomycin resistance in bacteria but may help to develop urgently needed therapeutic interventions.

## 2 Materials and Methods

### 2.1 Chemicals

The study utilized high-quality chemicals and antibiotics specifically designed for molecular biology and analytical purposes. The culture media brain heart infusion (BHI) broth, vancomycin, bacterial identification kit (HiStrep and HiStaph), ethidium bromide (EtBr), vancomycin Ezy MIC strip, teicoplanin disks, and Dodeca disks for testing antibiotic susceptibility were purchased from HiMedia, India. Lysozyme, proteinase K, and RNase were obtained from HiMedia, India, while Taq DNA polymerase and dNTPs were procured from Genei (Bengaluru, India). DNA ladders (BioLit ProxiB 100 bp and ProxiO 1 kb DNA ladder) were purchased from SRL, India, whereas oligonucleotides were obtained from Eurofins (Bengaluru, India). A Q5 high-fidelity DNA polymerase, a T4 DNA ligase, and restriction endonucleases (*Not*I and *Nde*I) were procured from New England Biolabs (Ipswich, MA, USA), while the plasmid vector pET22b(+) (Novagen) and a GenElute gel extraction kit were purchased from Sigma-Aldrich (Bengaluru, India). Luria–Bertani (LB) broth, tryptone, yeast extract, ampicillin, sodium acetate, sodium chloride, polyethylene glycol 8000 (PEG), sodium dodecyl sulfate (SDS), sodium hydroxide, magnesium chloride, imidazole, 4-(2-hydroxyethyl)-1-piperazine ethanesulfonic acid (HEPES) and 1,4-piperazinediethanesulfonic acid (PIPES) buffer, IPTG (isopropyl β-d-1-thiogalactopyranoside), and PMSF (phenylmethylsulfonyl fluoride) were purchased from HiMedia, India. Protein purification resin HisLink was procured from Promega (Madison, WI, USA), and protein molecular weight standards were procured from Thermo Fisher Scientific (Pierce Prestained Protein MW Marker). Bovine serum albumin (BSA), Coomassie brilliant blue R-250 (CBBR-250), glutathione, d-alanine, d-serine, ATP, dl-dithiothreitol, and Tris-HCL were purchased from HiMedia, India. d-Lactate, malachite green phosphate detection kit, and d-cycloserine were procured from Sigma-Aldrich (Bengaluru, India).

### 2.2 Isolation, Screening, Characterization, and Identification of Vancomycin-Resistant Bacteria

Vancomycin-resistant bacteria were screened from wastewater samples of the hospital in Kolhapur, MS, India (16.7021° N, 74.2267° E). From selected 10-fold serial dilutions, the 100 μL sample was spread plated on brain heart Iinfusion (BHI) agar with vancomycin (6 μg/mL) as per CLSI standards^37^ and incubated for 24 h at 37 °C. The vancomycin-resistant bacteria with distinct colony morphologies were selected, transferred on BHI-vancomycin agar slants, and preserved at 4 °C until further use. The screening process was further narrowed down based on the composition of the bacterial cell wall. The selected isolates were observed for their colony characteristics, and their morphology was examined using Gram staining. Bacterial identification kits HiStrep and HiStaph along with tests for pigment production and for hemolysis were employed to conduct a range of biochemical assays for biochemical characterization of the isolate. Furthermore, the data obtained from these assays were compared to information on similar bacterial strains reported in the literature to identify the specific bacterial strain.^22^ Tests for sugar fermentation including glucose, ribose, lactose, arabinose, sucrose, sorbitol, mannitol, raffinose, trehalose, and adonitol were conducted as per previous research by Manero and Blanch,^22^ whereas additional tests included arginine utilization, alkaline phosphatase, urease, malonate, lysine utilization, ornithine utilization, phenylalanine deamination, catalase, nitrate reduction, Voges–Proskauer, citrate utilization, and H_2_S production.^22^

The 16S rRNA gene amplification and sequencing method was used to identify the genotype of the selected isolate. Genomic DNA (gDNA) was extracted by a phenol-chloroform extraction method from the selected isolate.^38,39^ Briefly, bacterial cells were collected from 2 mL of an overnight shaking culture in BHI broth and then suspended in 460 μL of Tris-EDTA (TE) buffer containing proteinase K, lysozyme, and 10% (w/v) SDS (w/v). After 1 h of incubation at 37 °C, the bacterial cells were disrupted using double phenol-chloroform extractions, and total DNA was extracted by precipitation with a one/tenth volume of 3 M sodium acetate and a 0.6 volume of 100% isopropanol. The gDNA was solvated in nuclease-free water and quantified on a Nanodrop (BioSpectrometer fluorescence, Eppendorf). For the amplification of the 16S rRNA gene fragment, 15 pM samples of the universal forward 27F (CCA GAG TTT GAT CMT GGC TCA G) primer and the reverse 1492R (TAC GGY TAC CTT GTT ACG ACT T) primer were used in a 50 μL reaction that contained 100 ng of the DNA template, 200 μM dNTPs, 1.4 μL of a 5U Taq DNA polymerase, and 5 μL of 10× Taq buffer with 15 mM MgCl_2_. The thermal cycling conditions on a Mastercycler Nexus GX2 (Eppendorf) included an initial denaturation at 95 °C for 10 min; afterward, 40 cycles of denaturation at 95 °C for 1 min, annealing at 55 °C for 1 min, extension at 72 °C for 1 min, and a final extension step at 72 °C for 7 min were performed. The resulting amplicons were observed by agarose gel electrophoresis on 1% (w/v) agarose gel containing EtBr; after confirmation, amplicons were purified using PEG-NaCl precipitation^40^ and sequenced on an ABI 3730xl genetic analyzer using the BigDye Terminator v3.1 cycle sequencing kit. The 16S rRNA gene sequence obtained underwent comparison with the current GenBank nucleotide sequences database (https://blast.ncbi.nlm.nih.gov/) and the EzTaxon Database^41^ to identify closely related species. Reference sequences were selected based on BLASTn similarities. Subsequently, the 16S rRNA gene sequence and reference sequences were subjected to multiple sequence alignments using ClustalW, built-in MEGA11.^42^ The phylogenetic tree to infer the evolutionary history was constructed based on the neighbor-joining method in MEGA11,^42,43^ and the Kimura 2-parameter approach was utilized to calculate the evolutionary distances, which are expressed in base substitutions per site.^42,44^ The 16S rRNA gene sequence was then submitted to GenBank.

### 2.3 Assessment of Vancomycin Resistance and Its Mechanisms

The minimum inhibitory concentration (MIC) of vancomycin for the selected isolate was determined using both the microbroth dilution method^45^ in a 96-well plate and by an epsilometer test (E-test).^46^ The MICs were determined following the recommendations of the European Committee for Antimicrobial Susceptibility Testing (EUCAST, 2022; https://www.eucast.org/ast_of_bacteria/mic_determination) and the US Clinical and Laboratory Standards Institute^37^ using BHI broth supplemented with 50 mM d-Ser. An effective inoculum was prepared by inoculating a 24 h old culture of the selected isolate in BHI broth and incubating it at 37 °C until the turbidity equaled that of a McFarland standard of 0.5. The suspension was then diluted 1:100 to achieve a final bacterial count of 10^6^ bacterial cells/mL. Potential antimicrobial agents were prepared in a series of two-fold dilutions in BHI broth, and the final bacterial inoculum was diluted using an equal proportion of potential antimicrobial agents in a microwell plate. The plate was then incubated at 37 °C for 24 h, and the MIC was determined as the lowest concentration of a potential antimicrobial agent that prevented visible bacterial growth. For the E-test, a lawn culture of the selected test bacterial isolate was obtained by using a spread plate technique. Vancomycin Ezy MIC (HiMedia) strips with concentrations in the range of 0.016–256 μg/mL were placed on the plates, which were then incubated at 37 °C for 24 h. The MIC was determined at the intersection of the strip with a well-defined zone of inhibition.^46^

Following the CLSI guidelines^37^ and using the disk diffusion method, the selected isolate was also subjected to drug susceptibility tests for teicoplanin and 12 nonglycopeptide antibacterial drugs that included clarithromycin (15 μg/disk), gentamicin (10 μg/disk), streptomycin (10 μg/disk), nitrofurantoin (300 μg/disk), co-trimoxazole (25 μg/disk), amikacin (30 μg/disk), tobramycin (10 μg/disk), oxytetracycline (30 μg/disk), furazolidone (50 μg/disk), netillin (30 μg/disk), kanamycin (30 μg/disk), and nalidixic acid (30 μg/disk).

### 2.4 Detection of the Vancomycin Resistance Gene (*van* Gene)

The gDNA from the selected isolate was extracted using the well-established phenol-chloroform extraction method, as outlined in Section 2.2 as this method ensures the purity and integrity of the DNA.^38,39^ In the *van* gene detection study, we followed the established standard operating procedure, utilizing specific oligonucleotide primer sequences (refer to Table 1) and obeying the reaction conditions as outlined by the National Microbiology Laboratory in Winnipeg, Canada. The 25 μL PCR reaction on a MasterCycler Nexus GX2 (Eppendorf) comprised 100 ng of the DNA template, 12.5 pmol of each primer, 200 μM dNTPs, 2.5 μL of a 5U Taq DNA polymerase, and 2.5 μL of 10× Taq buffer with 15 mM MgCl_2_. The thermal cycling conditions included an initial denaturation at 95 °C for 15 min, followed by 35 cycles of denaturation at 94 °C for 1 min, annealing at a temperature specific for the respective primer (Table 1) for 1 min, extension at 72 °C for 1.5 min, and a final extension step at 72 °C for 7 min. After PCR, the amplified products were electrophoresed and visualized using a molecular imager Gel Doc XR+ imaging system (BioRad, U.S.). Subsequently, we sequenced and analyzed them using the BLASTn Search Tool^47^ to confirm the gene.

**Table 1:** Primers Used in the *van* Gene Detection Study^a^

encoding gene	primer name	primer sequence	product size	description of the encoded enzyme	annealing temperature in °C
*vanC1*	VANC1	GAAAGACAACAGGAAGACCGC	796	d-alanine-d-serine ligase	55
	VANC2	ATCGCATCACAAGCACCAATC			
*vanA*	VANA1	GGGAAAACGACAATTGC	732	d-alanine-d-lactate ligase	45
	VANA2	GTACAATGCGGCCGTTA			
*vanB*	VANB1	AAGCTATGCAAGAAGCCATG	538	d-alanine-d-lactate ligase	50
	VANB2	CCGACAATCAAATCATCCTC			
*vanD*	Duni1	TTATATYGGRATYACAAAATC	628	d-alanine-d-lactate ligase	44
	Duni2	CTGYGCTTCCTGRTGRATCTT			
*ddl*	ddlEfs-1	TTATTTTGTTGTATGGCGGC	947	d-alanine-d-alanine ligase	48
	ddlEfs-2	AAAGTCAGTAAAACCAGGCA			
*ddl*	ddlEfm-1	ATTACAAAGGCAGAAAACCG	333	d-alanine-d-alanine ligase	49
	ddlEfm-2	TGTCAAAAAGAAATCGCACC			
*vanM*	vanMF	GATAATGAACACTGTCGCTC	810	d-alanine-d-lactate ligase	49
	vanMR	AATTGTTATACCTGCTGAGAC			
*vanC3*	VANC3A	GCCTTTACTTATTGTTCC	224	d-alanine-d-serine ligase	44
	VANC3B	GCTTGTTCTTTGACCTTA			
*vanN*	vanN1	CTAGYGTYTTGTCTGTATTAGA	923	d-alanine-d-serine ligase	50
	vanN2	CTGATAAGTGATRCCYGATGC			
*vanL*	vanL1	TCTCACCACAAGCAACAAC	718	d-alanine-d-serine ligase	50
	vanL2	GTCCTGAACAGCCTAGTAAC			
*vanE*	vanE1	TGTGGTATCGGAGCTGCAG	513	d-alanine-d-serine ligase	48
	vanE2	GTCGATTCTCGCTAATCC			
*vanG*	vanG5	TTCGATTTCATCAACTCTGC	374	d-alanine-d-serine ligase	48
	vanG6	CAGGAATACCTGTTGTTGG			

a These primers were designed by the National Microbiology Laboratory, Winnipeg, Canada.

### 2.5 Expression and Purification of the vanC2 d-Ala-d-Ser Ligase

#### 2.5.1 Construction of the Expression Vector for vanC2 EcfDdls of *E. casseliflavus*

The *vanC2* gene sequence of *Enterococcus casseliflavus* CCM 439 (accession no. NG_048344)^48^ served as the reference sequence for primer design to amplify the *vanC2* gene from the selected isolate. Primers were designed using the Primer3 server.^49^ The forward primer (GGG GGG CAT ATG AAA AAA ATC GCC ATT ATT TTT GGA GGC) contained an *Nde*I site (underlined), and the reverse primer (GAG CGG CCG CAA ATT TGA CTT CCT CCT TTG C) contained an *Not*I site (underlined). These primers were selected to enable the seamless insertion of a 6× His tag site from the pET22b(+) expression system to the C-terminus of the target *vanC2* gene. Furthermore, a comprehensive *in silico* cloning simulation was carried out to verify the successful insertion of the *vanC2* gene into pET22b(+) using advanced SnapGene software (www.snapgene.com) (Figures S1 and S2).

The pET22b-vanC2 recombinant vector was engineered by inserting the purified *vanC2* gene into the pET22b(+) vector. To enhance their transformation efficiency, both *Escherichia coli* (*E. coli*) DH5α and *E. coli* BL21(DE3)pLysS strains underwent a rigorous process to render them ultracompetent using the Inoue technique.^50^ Subsequently, the ultracompetent *E. coli* strain DH5α was employed for cloning. Verification of the desired *vanC2* gene insertion into the recombinant clone was accomplished through restriction digestion utilizing *Nde*I and *Not*I restriction enzymes, followed by detection of the *vanC2* gene using PCR. Afterward, the sequence-confirmed recombinant pET22b-vanC2 plasmid was transformed into ultracompetent *E. coli* BL21(DE3)pLysS for the expression of the gene encoding a protein tagged with 6× His at the C-terminus to facilitate affinity chromatography.

#### 2.5.2 Overexpression, Purification, and SDS-PAGE Analysis of the vanC2 d-Ala-d-Ser Ligase

*E. coli* BL21(DE3)pLysS cells harboring the pET22b-vanC2 vector were cultivated at 37 °C in 1 L of LB broth supplemented with ampicillin until the optical density at 600 nm reached 0.6. Subsequently, 500 μL of 1 M IPTG was added, and the culture was further incubated for 6 h at 20 °C with vigorous shaking to induce the synthesis of the recombinant d-Ala-d-Ser ligase. Unless stated otherwise, all subsequent procedures were conducted at 4 °C. Following induction, cells were centrifuged (8000 rpm, 10 min) in 50 mL aliquots. The cells from each 50 mL culture were resuspended in 5 mL of lysis buffer A composed of 50 mM HEPES, 5 mM MgCl_2_, 10 mM imidazole, 10% glycerol, and 1 mM PMSF (pH 8.0) and then disrupted by probe sonication (6 cycles of a 20 s pulse with 10 s cooling intervals). Cell lysates were centrifuged at 10,000 rpm for 30 min to collect the supernatant, which was subsequently applied to a Ni-NTA column pre-equilibrated with buffer A without PMSF. After washing the column with the same buffer, EcfDdls was eluted using a 10 mL linear gradient of 50–500 mM imidazole in buffer B (50 mM HEPES, 5 mM MgCl_2_, imidazole, 10% glycerol, and 300 mM NaCl, pH 8.0).

The eluate was dialyzed twice for 2 h and overnight against a 10-fold larger volume of buffer C (50 mM HEPES, 150 mM KCl, 5 mM MgCl_2_, 5 mM glutathione, and 20% glycerol, pH 7.2). Protein quantification was performed using the Bradford assay with BSA as a reference. To assess the purity of the His-tagged protein, samples were resolved on SDS-PAGE according to the protocol by Laemmli and stained with Coomassie brilliant blue (R-250), and the molecular weight of denatured EcfDdls was estimated by comparison with Pierce Prestained Protein MW Markers (Thermo Fisher Scientific India Pvt. Ltd.). Additionally, the molecular weight of the d-Ala-d-Ser ligase, encoded by the reference *vanC2* gene of *E. casseliflavus* CCM 439, was predicted using the ExPASy ProtParam server^51^ (accession no. NG_048344).

### 2.6 Assessment of EcfDdls Activity and Its Kinetics

The activity of the enzyme EcfDdls was assessed using the colorimetric malachite green method,^52^ which quantifies the release of inorganic phosphate during the reaction (Figure S3A). The enzyme’s activity was measured for 30 min in a reaction with a total volume of 50 μL at 37 °C in a 96-well microwell plate containing 20 mM Tris-HCl (pH 8.0), 0.2 μg of pure EcfDdls, 10 mM KCl, 10 mM MgCl_2_, 1 mM DTT, 100 μM ATP, 1 mM d-Ala, and 1 mM d-Ser. All assay components were preincubated at 37 °C for 30 min before the addition of a substrate and a cofactor. The reactions were initiated by adding substrates (d-Ala and d-Ser) and a cofactor (ATP) to the preincubated reaction mixture. The resultant reaction mixture was further incubated for 30 min at 37 °C. The concentration of released inorganic phosphate was determined by adding a malachite green reagent (malachite green phosphate detection kit, Sigma-Aldrich, Bengaluru, India) according to the manufacturer’s instructions. The absorbance was recorded at 630 nm by using a microplate reader (BioTek 800 TS microplate reader).

The optimal pH of EcfDdls was ascertained by incubating the enzyme in a reaction mixture similar to that used in the functional assay and varying pH levels to 7, 7.2, 7.5, 8.0, 8.2, and 8.5 using 50 mM HEPES-NaOH buffer. The concentration of released inorganic phosphate at different pH conditions was determined by adding a malachite green reagent and measuring the absorbance at 630 nm. The thermal stability of EcfDdls was determined by measuring the enzyme activity in the HEPES-NaOH buffer (pH 8.0) at different temperatures (4, 15, 30, 37, 42, and 55 °C) while maintaining the same concentration of the reaction components as in the enzyme activity test. The concentration of released inorganic phosphate under various temperature conditions was determined by adding a malachite green reagent and monitoring the absorbance at 630 nm.

The specificity of EcfDdls for substrates d-Ala, d-Ser, and d-Lac at subsite 1, i.e., the first substrate binding site, and subsite 2, i.e., the second substrate binding site, was determined by detecting released inorganic phosphate with a malachite green reagent. For this purpose, 10 mM substrates d-Ala, d-Ser, and d-Lac were added to the reaction mixture containing similar components as in the functional assay to detect d-Ala-d-Ala, d-Ser-d-Ser dipeptide, and d-Lac-d-Lac dipeptide, respectively. Furthermore, the addition of a 10 mM amino acid substrate to the respective reaction mixture allowed us to pinpoint the existence of the d-Ala-d-Ser dipeptide and the d-Ala-d-Lac depsipeptide.

An inorganic phosphate detection assay was used to analyze the kinetic parameters of EcfDdls. The Michaelis constant (*K*_m_) and maximal reaction velocity (*V*_max_) of d-Ala, d-Ser, and ATP were calculated. Enzymatic reactions were carried out at different d-Ala concentrations with constant d-Ser and ATP. Then, the d-Ser concentrations were varied while maintaining constant concentrations of d-Ala and ATP and likewise for ATP concentrations. This helped to determine their respective *K*_m_ and *V*_max_ values. Every enzymatic reaction in this investigation was carried out in triplicate. The background absorbance of the reaction mixture without EcfDdls was measured and deducted from the absorbance values with EcfDdls to remove the interference caused by the buffer components. The *K*_m_ and *V*_max_ values were calculated using nonlinear regression of the Michaelis–Menten model in GraphPad Prism 5.0 (GraphPad Software, Inc., San Diego, CA, United States). Additionally, the turnover number (*k*_cat_) was determined by using the molecular weight of EcfDdls (39 kDa).

### 2.7 Assessment of Inhibition of *E. casseliflavus* by a Virtually Screened Inhibitor

**Figure 1:**
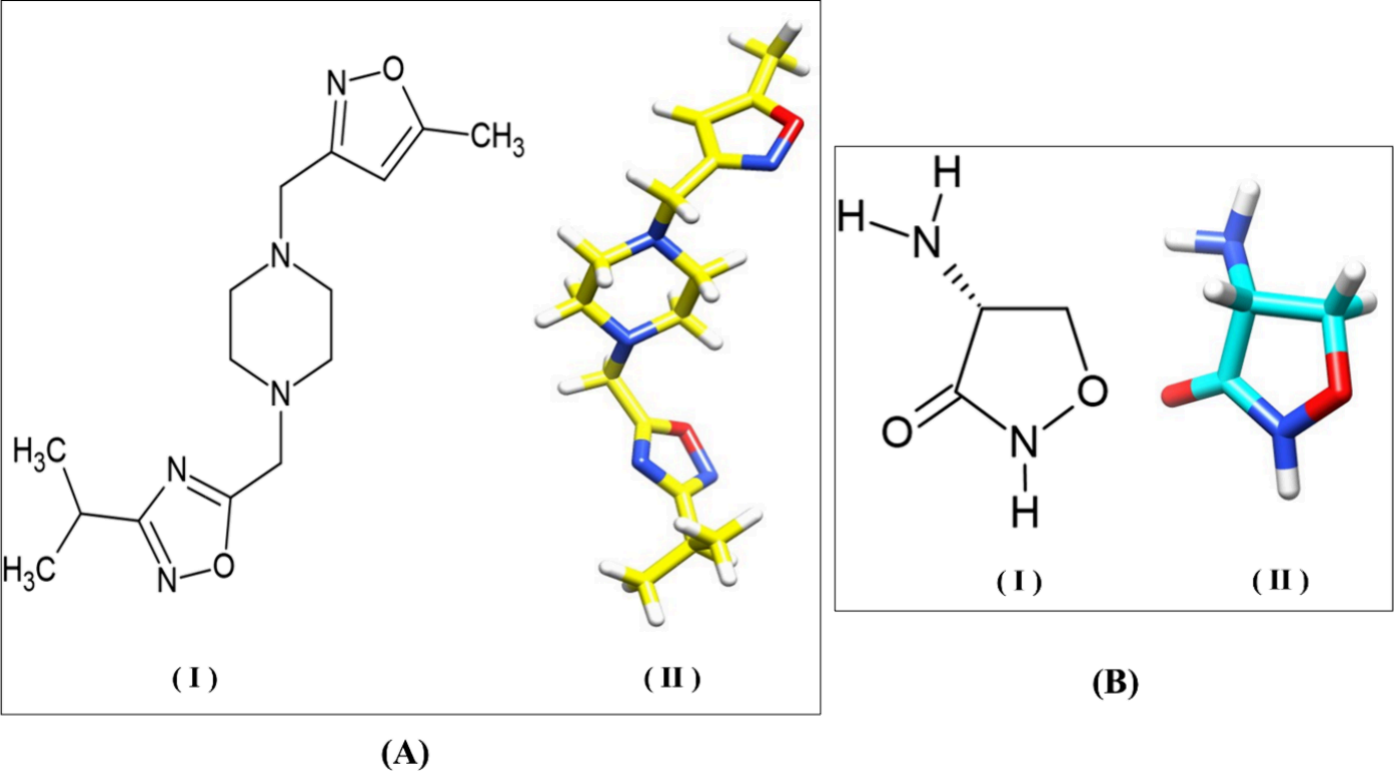
Two- and three-dimensional structure of (A) virtually screened oxadiazole derivative CID 45805715 and (B) known inhibitor DCS. The two- and three-dimensional structures are generated by using ChemSketch version 2024.2.0 from ACD/Laboratories, Toronto, ON, Canada, www.acdlabs.com, and UCSF Chimera.^56^

One of the potent virtually screened inhibitors, 1-[(5-methyl-1,2-oxazol-3-yl)methyl]-4-{[3-(propan-2-yl)-1,2,4-oxadiazol-5-yl]methyl}piperazine (referred to as CID 45805715) (Figure 1A), investigated in an earlier study,^53^ was procured from Enamine Ltd., Ukraine, through Sigma-Aldrich (Bengaluru, India) to examine *in vitro* enzymatic inhibition of purified EcfDdls as well as microbiological evaluation. To assess the inhibitory properties of CID 45805715, we conducted *in vitro* enzymatic inhibition studies using a malachite green reagent-based inorganic phosphate detection assay and compared it to DCS (Figure 1B), a known Ddl inhibitor.^54^ The inhibitory effect of CID 45805715 and DCS was evaluated at 500 μM for their capacity to impede EcfDdls activity. Prior to the assay, the enzyme and inhibitor were preincubated for 30 min at 37 °C. After preincubation, substrates d-Ala and d-Ser and cofactor ATP were added, and the reaction mixture was incubated for another 30 min at 37 °C. The final reaction mixture (50 μL) contained 20 mM Tris-HCl (pH 8), 10 mM MgCl_2_, 10 mM KCl, 1 mM DTT, 100 μM ATP, 500 μM inhibitor, 1 mM d-Ala, 1 mM d-Ser, and 0.2 μg of purified EcfDdls. Both CID 45805715 and DCS were dissolved in 5% DMSO for the experiments.

The experiment involved determining the IC_50_ values for CID 45805715 and DCS under conditions similar to those used in the inhibition study. The CID 45805715 compound and DCS were tested at various concentrations. Furthermore, the MIC values for the screened compounds CID 45805715 and DCS were determined using the microbutter dilution method as outlined in Section 2.3. A final concentration of 1% DMSO was used, based on the findings of Ameryckx et al.,^55^ which showed that a 1% DMSO concentration did not affect bacterial growth.

## 3 Results

### 3.1 Isolation, Screening, Characterization, and Identification of Vancomycin-Resistant Bacteria

Hospital effluent wastewater is anticipated to harbor a substantial concentration of antibiotic-resistant bacteria and resistance-instigating genes, given the high levels of antibiotic usage in these environments. Therefore, in the present study, hospital wastewater samples were subjected to microbiological screening for bacterial colonies on brain heart infusion (BHI) agar supplemented with vancomycin. Following incubation at 37 °C for 24 h, uniculture colonies of vancomycin-resistant bacteria were observed on the BHI-vancomycin agar plates, where serial dilutions of hospital wastewater samples had been applied. Initially, 120 bacterial strains exhibiting distinct morphologies were meticulously isolated as pure cultures in primary screening. Subsequent screening, based on peptidoglycan composition, facilitated the identification of the Gram-positive isolate S6 for detailed investigation (Figure S4). Cultivation of the vancomycin-resistant isolate S6 on BHI-vancomycin agar unveiled its unique growth pattern, manifesting as circular, yellow-colored colonies measuring less than 1 mm in diameter (Figure S4A). Microscopic examination employing Gram staining elucidated the morphological characteristics of isolate S6 as Gram-positive cocci, exhibiting active motility under light microscopy. The selected vancomycin-resistant isolate S6 was characterized by using conventional biochemical techniques, and further identification was accomplished through 16S rRNA gene sequencing. Notably, these cocci demonstrated the ability to hydrolyze bile-esculin and l-pyrrolidonyl-β-naphthylamide, to produce a yellow pigment along with robust growth observed at 7.5% NaCl concentrations (Table 2). Conversely, S6 exhibited negative responses to Voges–Proskauer, alkaline phosphatase production, catalase production, citrate utilization, H_2_S production, arginine utilization, phenylalanine deamination, and nitrate reduction tests. Acid production, indicative of sugar metabolism diversity, was evident. Detailed biochemical characterization findings are given in Table 2. Gram staining further revealed the spatial arrangement of cocci, occurring predominantly in pairs, tetrads, or clusters, rather than forming chains (Figure S4B).

**Table 2:** Phenotypic and Biochemical Characterization of the Vancomycin-Resistant Isolate S6^a^

microscopic observations	
Gram’s nature	positive
morphology	cocci
arrangements	pairs, tetrads, or clusters
motility	actively motile

colony characteristics	
size	<1 mm
shape	circular
surface	smooth
margin	entire
elevation	raised
color	yellow
opacity	opaque

biochemical characterization	results
Voges–Proskauer’s	-
esculin hydrolysis	+
PYR	+
ONPG	-
arginine utilization	-
glucose	+
lactose	+
arabinose	+
sucrose	+
ribose	+
sorbitol	+
mannitol	+
raffinose	+
alkaline phosphatase	-
urease	-
trehalose	+
maltose	+
malonate	+
citrate	-
nitrate reduction	-
catalase	-
lysine	-
ornithine	-
phenylalanine deamination	-
H_2_S production	-
adonitol	+
pigment	yellow
hemolysis	α-hemolysis

a Note: +, positive; -, negative.

**Figure 2:**
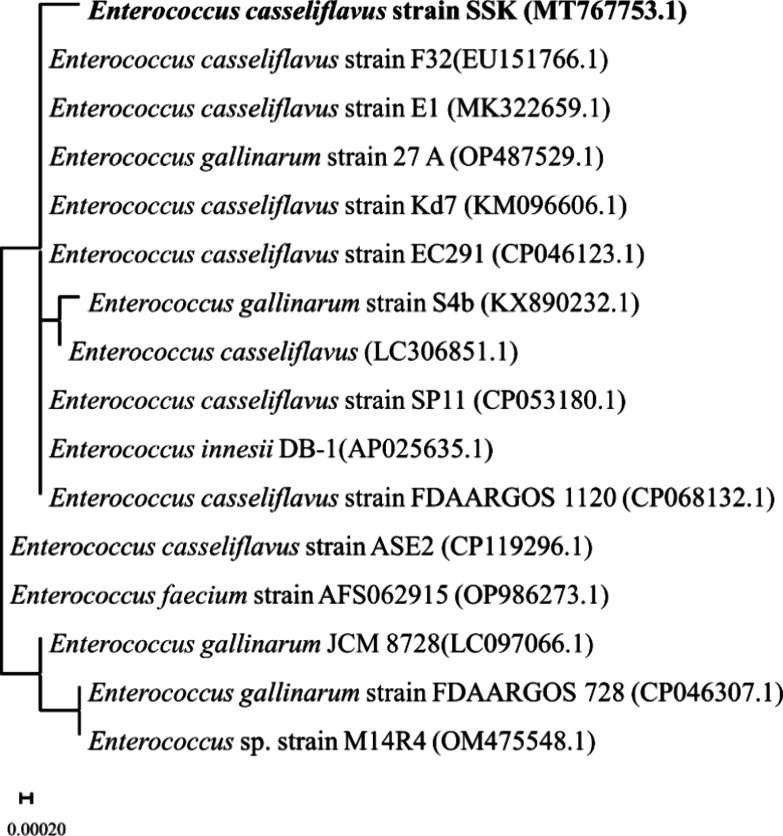
Evolutionary relationship of *E. casseliflavus* SSK based on the 16S rRNA gene and closely related species of Enterococcus. The phylogenetic tree was constructed by the neighbor-joining method by using MEGA11 software (the scale represents 0.0002 nucleotide substitution).

Subsequently, PCR amplification and sequencing of the 16S rRNA gene were performed, followed by sequence similarity analysis using BLASTn. The results revealed that isolate S6 shows a high degree of similarity to the 16S rRNA gene of bacteria belonging to the *Enterococcus casseliflavus* species. The 16S rRNA gene sequence spanning 1392 base pairs extracted from isolate S6, along with closely related species, served as the foundation for constructing a phylogenetic tree (Figure 2 and Figure S5). Notably, isolate S6 demonstrated the highest sequence similarity with strains of *E. casseliflavus* strain FDAARGOS 1120, *E. innesii* DB-1, and *E. casseliflavus* strain SP11, as evidenced by the neighbor-joining phylogeny tree. Furthermore, a comparative analysis of the 16S rRNA gene sequence of isolate S6 with entries in the EzTaxon Database^41^ revealed its affiliation with a distinct clade, tightly clustered with strains of *E. casseliflavus* and *E. gallinarum* (Figure 2). Consequently, the isolate was unequivocally identified as a novel bacterial strain designated as *E. casseliflavus* SSK, with its 16S rRNA gene sequence deposited in GenBank under accession number MT767753.1.

### 3.2 Assessment of Vancomycin Resistance and Its Mechanisms

**Figure 3:**
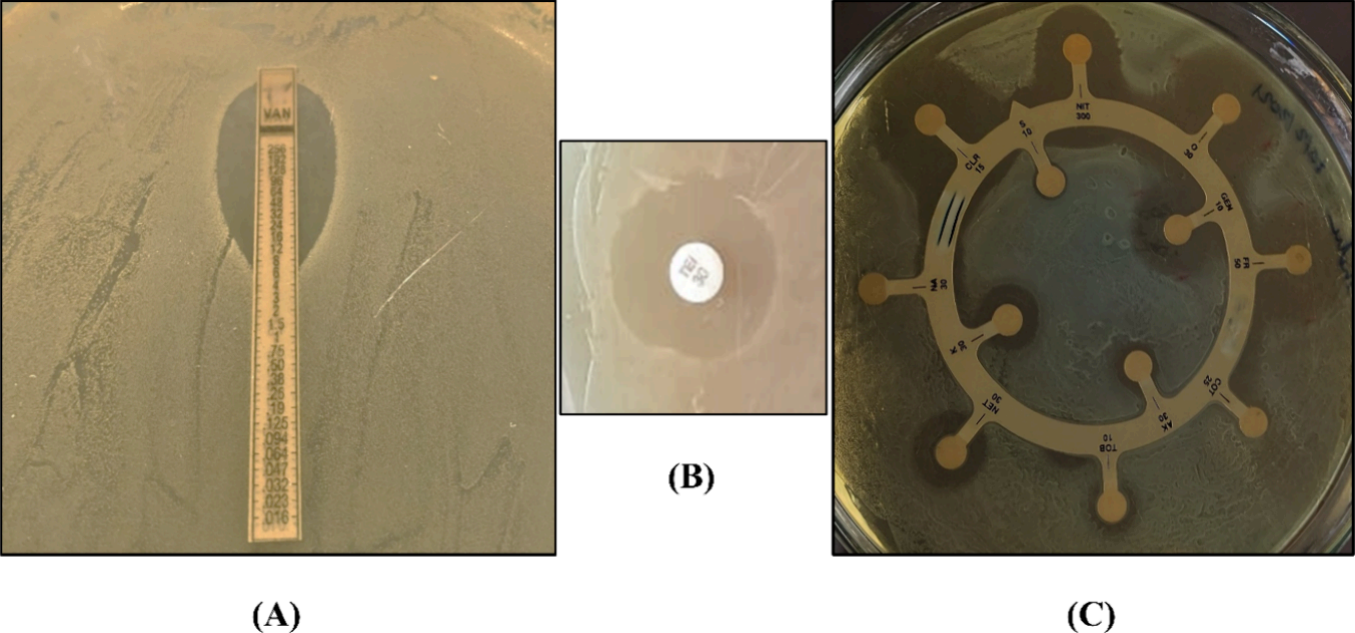
(A) Determination of the minimum inhibitory concentration for vancomycin. The E-test inhibition zone indicates the MIC at 8 μg/mL. The strip has a vancomycin concentration of 0.016–256 μg/mL. (B) Zone of inhibition around the teicoplanin disk (30 μg/mL) indicating sensitivity of *E. casseliflavus* SSK for teicoplanin and (C) antibiotic susceptibility test results showing susceptible and resistant zones of isolate *E. casseliflavus* SSK.

To elucidate the mechanisms underlying drug resistance, we conducted comprehensive assessments on isolate S6. The minimum inhibitory concentration (MIC) of vancomycin for isolate S6 was meticulously determined using brain heart infusion (BHI) broth supplemented with 50 mM d-Ser, a crucial cofactor known to influence resistance induction rates, as per Dutta and Reynolds.^57^ This investigation conclusively determined a minimum inhibitory concentration (MIC) value of 8 μg/mL for vancomycin using the micro broth dilution method, which was further validated by an E-test. Following 24 h of incubation at 37 °C, the BHI agar plate inoculated with isolate S6 exhibited a distinct and well-defined zone of inhibition, with the intersection of the vancomycin strip occurring at a concentration of 8 μg/mL (Figure 3A). The comprehensive findings emphasize the isolate’s resistance to streptomycin, co-trimoxazole, and nalidixic acid, which belong to the aminoglycoside, sulfonamide, and quinolone classes of antibiotics, respectively, while also displaying intermediate resistance to several aminoglycosides including amikacin, tobramycin, netillin, and kanamycin (Figure 3C and Table S1). However, the isolate retains sensitivity to teicoplanin (Figure 3B).

### 3.3 Detection of the Vancomycin Resistance Gene (*van* Gene)

**Figure 4:**
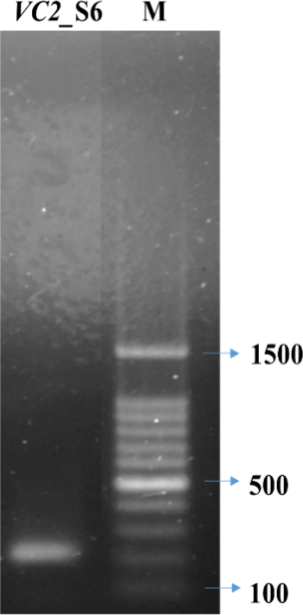
*vanC2* gene detection by PCR from vancomycin-resistant isolate S6; M, molecular size marker (BioLit ProxiB 100 bp DNA ladder, SRL, India).

The molecular analyses suggest that isolate S6 potentially harbors the vanC2-type vancomycin resistance gene, as indicated by PCR studies (Figure 4). The nucleotide sequence of the *van* gene from isolate S6 demonstrated a 100% match to the corresponding vanC2/3 sequence from *E. casseliflavus* CCM 439 (accession no. NG_048344). Thus, sequencing and similarity search analyses corroborated these findings, identifying the amplified product as the *vanC2* gene, known to encode the d-Ala-d-Ser ligase responsible for intrinsic vancomycin resistance in *E. casseliflavus*.^58^

### 3.4 Expression and Purification of the vanC2 d-Ala-d-Ser Ligase

To further elucidate the molecular basis of resistance, we employed a series of molecular biology techniques. The *vanC2* gene was meticulously amplified, purified, and successfully cloned into the overexpression vector pET22b(+), enabling robust expression of the d-Ala-d-Ser ligase from *E. casseliflavus* strain SSK. The presence of the *vanC2* gene within the construct was confirmed by the release of an approximately 1100 bp insert in restriction mapping analysis (Figure S6A), and its integrity was validated through PCR-based gene identification (Figure S6B). The partial sequence reveals 98.94% identity with the *vanC2/3* gene for the d-Ala-d-Ser ligase in *E. casseliflavus* UC73 (accession no. NG_048350.1).

**Figure 5:**
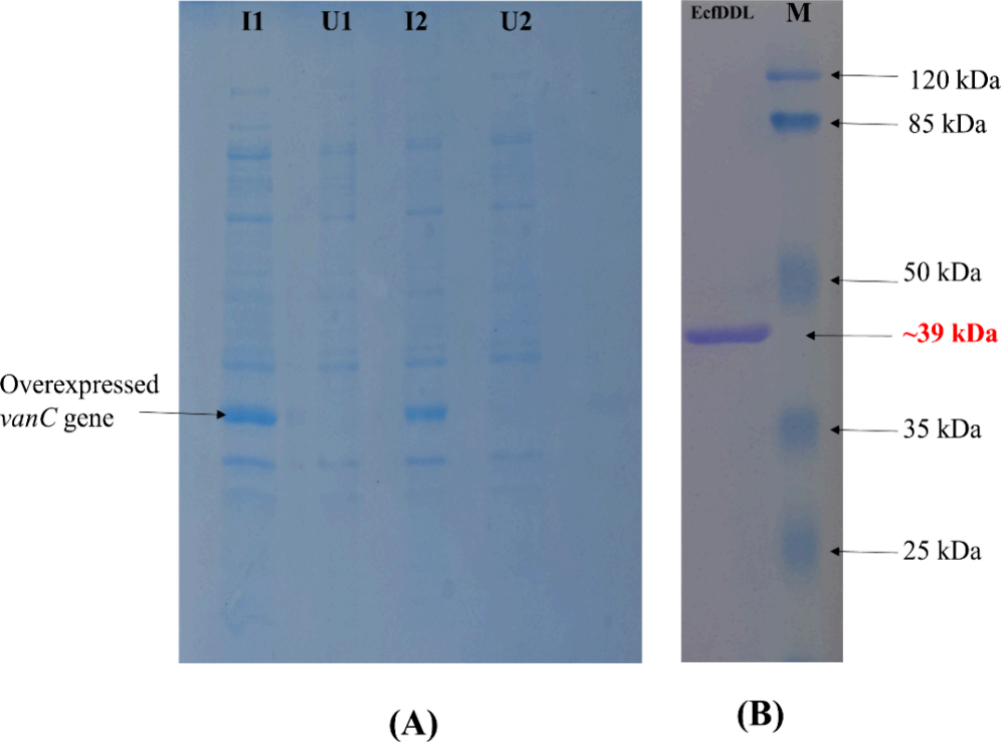
Analysis of EcfDdls fusion protein by SDS-PAGE. (A) SDS-PAGE analysis of induced and uninduced *E. coli* cell lysates. (B) Purified EcfDdls protein (with the 6× histidine tag) showing a molecular weight ∼39 kDa with a single intact band (lane EcfDdls); the molecular weight of the purified enzyme was determined by comparing with standard molecular weight markers (Thermo Fisher Scientific Pierce Prestained Protein MW Marker) of 25, 35, 50, 85, and 120 kDa (from bottom to top).

Following transformation into *E. coli* strain BL21(DE3) pLysS cells, the recombinant pET22b-vanC2 construct facilitated robust expression of the C-terminal His-tagged EcfDdls protein. The SDS-PAGE analysis confirmed successful expression and purification of the fusion protein, achieving >90% purity (Figure 5A,B), with a molecular mass of approximately 39 kDa.

### 3.5 Assessment of EcfDdls Activity and Its Kinetics

**Figure 6:**
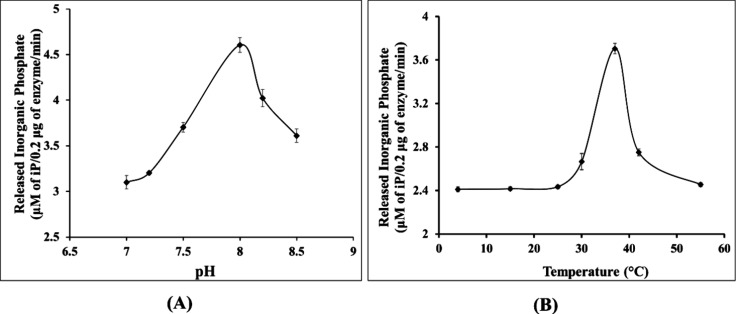
Effects of pH (A) and temperature (B) on EcfDdls activity. (A) For pH profile analysis, the activity of EcfDdls was measured at different pH using HEPES-NaOH buffer at 37 °C. (B) For evaluating the influence of temperature on EcfDdls activity, the enzymatic activity was processed from 4 to 55 °C in the HEPES-NaOH buffer (pH 8.0).

The enzymatic activity of EcfDdls was meticulously assessed through a colorimetric assay based on liberated inorganic phosphate from ATP hydrolysis, indicative of d-Ala-d-Ser ligase activity (Figure S3B). The results demonstrate robust enzyme activity, with 0.5 μM inorganic phosphate released per min by 0.2 μg of EcfDdls. Optimal enzymatic activity was observed at pH 8, while the temperature profile revealed enhanced activity between 30 and 37 °C (Figure 6A,B), underscoring the functional integrity of the purified protein.

**Figure 7:**
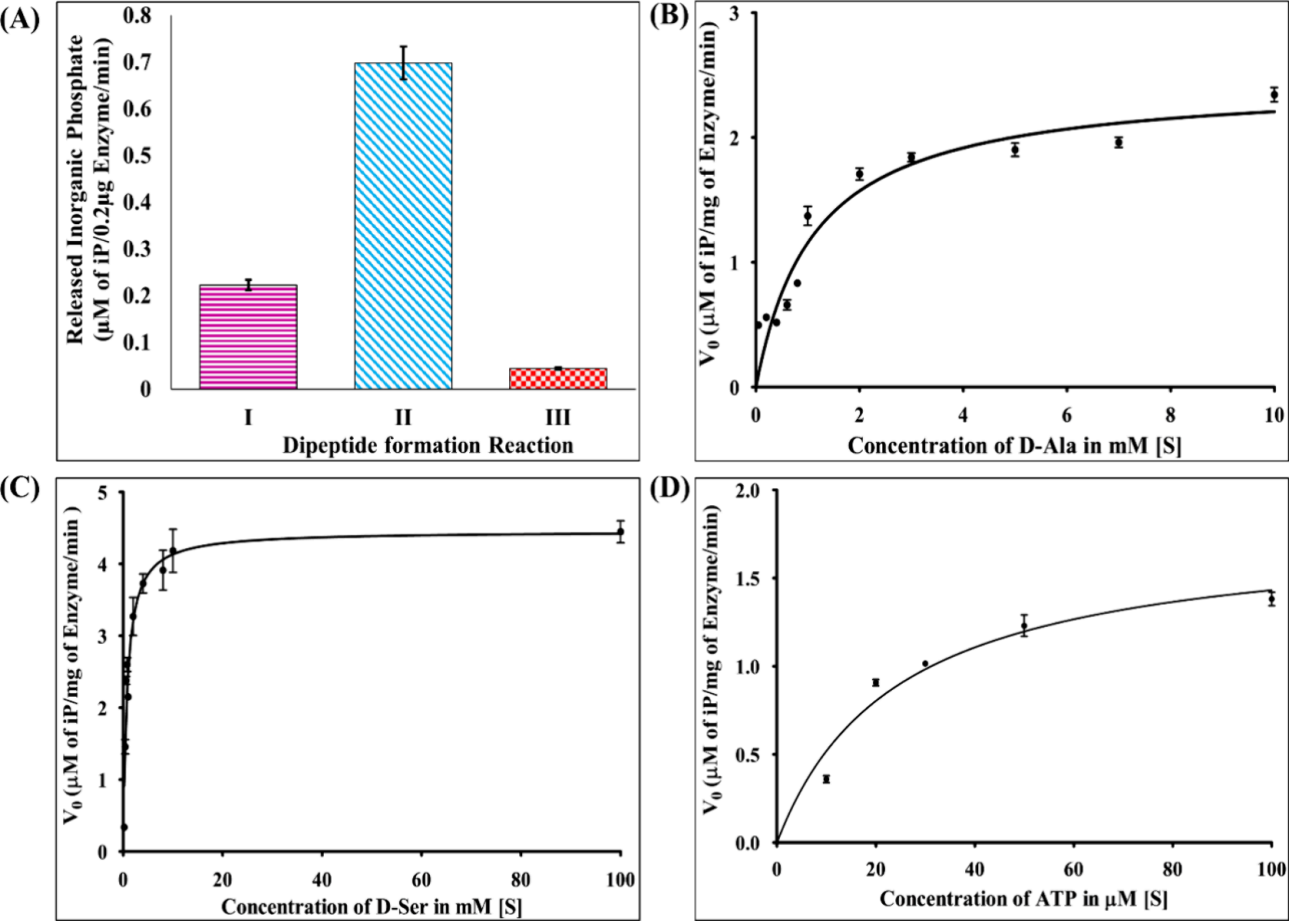
Substrate specificity and kinetic analysis of EcfDdls. (A) Analysis of substrate specificity of EcfDdls (I) for formation of the d-Ala-d-Ala dipeptide, (II) for formation of the d-Ala-d-Ser dipeptide, and (III) for formation of the d-Ala-d-Lac dipeptide. (B) *K*_m1_ of d-Ala for subsite 1. (C) *K*_m2_ of d-Ser for subsite 2. (D) *K*_m_ of ATP.

The substrate specificity of EcfDdls was explored through the detection of inorganic phosphate during the dipeptide formation reaction, revealing notable trends (see Figure 7A). Specifically, a substantial release of inorganic phosphate was observed in the presence of d-Ser and d-Ala, indicative of robust enzymatic activity. Furthermore, the presence of d-Ala alone also elicited a discernible release of inorganic phosphate. Conversely, when d-Ala was combined with d-Lac, only trace amounts of inorganic phosphate were detected.

To elucidate the kinetic behavior of EcfDdls, reaction rates were assessed across varying substrate concentrations, yielding critical enzymatic parameters (Figure 7B–D). The *K*_m1_ value for d-Ala at subsite 1 and the *K*_m2_ value for d-Ser at subsite 2 were determined to be 1.12 ± 0.1 and 0.8 ± 0.1 mM, respectively, underscoring the affinity of the enzyme for these substrates. Additionally, the *K*_m_ value for ATP was found as 24.31 ± 4.1 μM. Complementary to these findings, the corresponding *V*_max_ values were calculated as 0.024 ± 0.1 mM min^–1^ for d-Ala, 0.044 ± 0.1 mM min^–1^ for d-Ser, and 1.77 ± 0.11 μM min^–1^ for ATP. Notably, the catalytic efficiency of EcfDdls toward d-Ser, represented by the *K*_cat_ value of 364.8 min^–1^, exemplifies the enzyme’s robust performance.

### 3.6 Assessment of Inhibition of *E. casseliflavus* by a Virtually Screened Inhibitor

**Figure 8:**
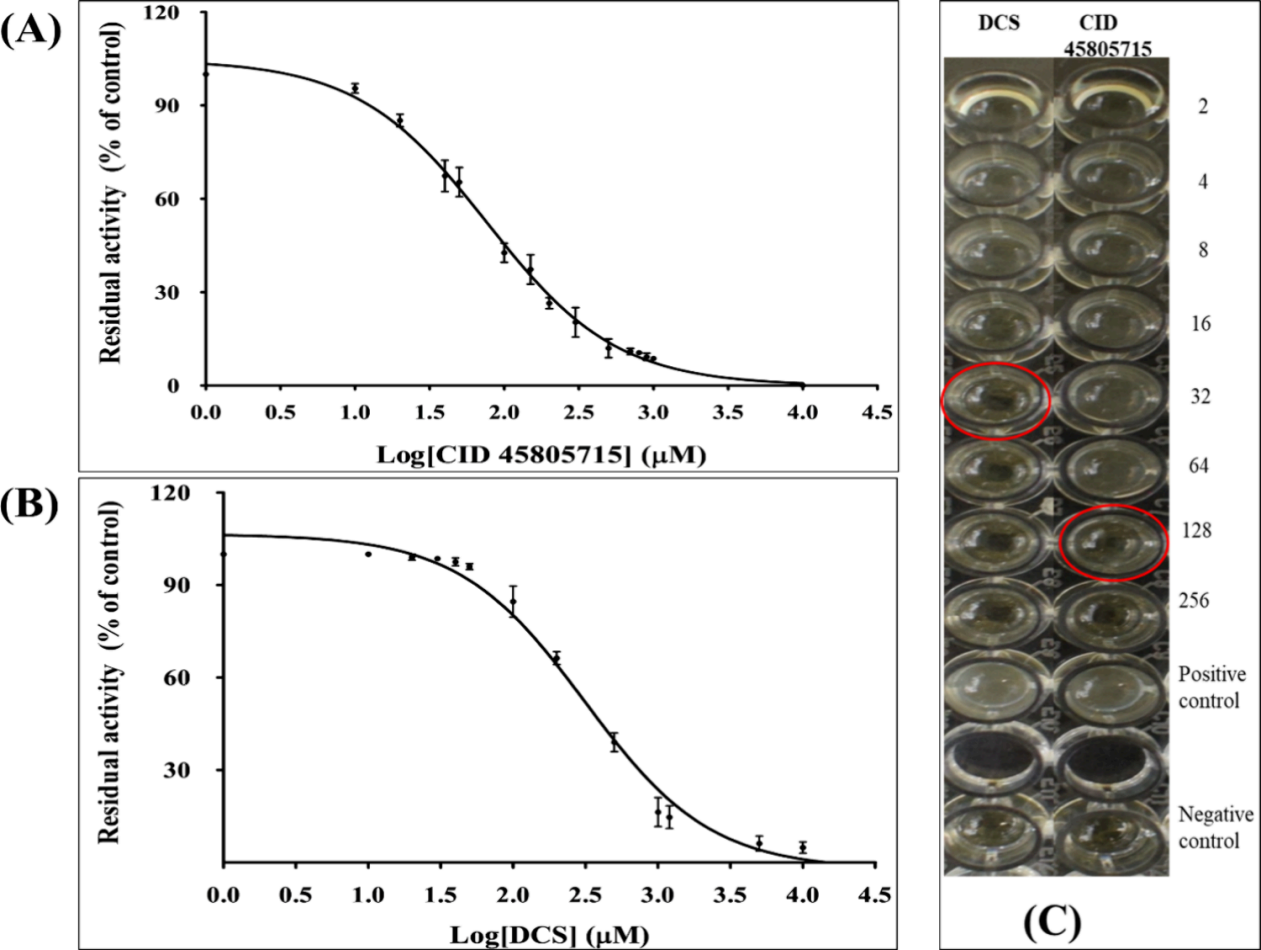
Inhibition of EcfDdls and *E. casseliflavus* strain SSK by a virtually screened oxadiazole derivative and known inhibitor DCS. Estimation of IC_50_ values is in GraphPad Prism 5.0. Residual activity (% of the control) is plotted on the vertical axis and log (concentration) in μM on the horizontal axis. The IC_50_ is the concentration at which the curve passes through the 50% inhibition level. (A) IC_50_ analysis for CID 45805715, (B) IC_50_ analysis for DCS, and (C) MIC values for the virtually screened inhibitor CID 45805715 (128 μg/mL) and DCS (32 μg/mL).

The half-maximal inhibitory concentration value (IC_50_) was determined by fitting the residual activity value in percentage as a function of respective inhibitor concentration using a nonlinear regression algorithm for log(concentration) versus response, built into GraphPad Prism version 5.00 (for Windows, GraphPad Software, San Diego, California, USA, www.graphpad.com). The IC_50_ of CID 45805715 was quantified as 76.7 ± 1.01 μM, while the reference compound DCS exhibited an IC_50_ of 313 ± 1.6 μM (see Figure 8A,B). Furthermore, the minimum inhibitory concentration (MIC) shed light on the potency of the virtually screened inhibitor CID 45805715 against *E. casseliflavus* strain SSK, revealing a value of 128 μg/mL (Figure 8C). In comparison, DCS demonstrated an MIC value of 32 μg/mL, indicating its relatively higher potency.

## 4 Discussion

The bacterial cell wall has long been recognized as a prime target for antimicrobial drugs due to its unique presence in prokaryotic cells and its vital role in bacterial survival.^59^ The peptidoglycan, a key constituent of the cell wall, is already targeted by two major therapeutic groups, β-lactams and glycopeptides, which inhibit the late stages of peptidoglycan synthesis.^59^ However, the emergence of resistance to these antibiotics has revealed the remarkable flexibility of this pathway.^60^ With escalating resistance to vancomycin, it is imperative to identify alternative targets within the peptidoglycan synthesis pathway.^4,61^ Despite this urgency, many enzymes involved in the early stages of peptidoglycan production remain underexplored.^62−64^ The Ddl enzyme, utilizing essential bacteria-specific d-amino acid substrates, is particularly interesting in this context.^65^ Notably, vancomycin-resistant Gram-positive bacteria possess similar enzymes that employ different substrates, such as d-Lac or d-Ser.^5,66,67^

Vancomycin’s effectiveness is due to its ability to bind firmly to the bacterial cell wall precursor lipid II, hindering cell wall production through a network of five hydrogen bonds with the d-Ala-d-Ala terminus of the lipid II stem pentapeptide.^4,68−70^ This interaction sequesters lipid II and sterically inhibits the subsequent stages of transglycosylation and transpeptidation, thereby inhibiting cell wall production.^4,68−70^ The activity of glycopeptides ultimately causes the cell wall to become unstable, leading to bacterial cell death, likely due to osmotic stress. The selective activity of vancomycin against Gram-positive organisms was explained by their display of peptidoglycan precursors on the surface of their cytoplasmic membrane, while Gram-negative bacteria are shielded by an outer lipopolysaccharide barrier.^71−73^ Consequently, the focus pivoted toward investigating a vancomycin-resistant Gram-positive isolate.

The classic biochemical characterization of Enterococcus strains, distinguishing them from other similar genera, involves species identification. Catalase-negative Gram-positive cocci that thrive in a 6.5% NaCl medium are presumed to be members of the Enterococcus genus.^74^ The catalase production test was pivotal, revealing the isolate’s affiliation with the Enterococcus family.^22,75^ However, the production of yellow pigments and ribose utilization differentiate *E. casseliflavus* SSK from *E. gallinarum* and *E. flavescens*.^22^ Vancomycin resistance investigations using both the micro broth dilution method and E-test uncovered the presence of low-level vancomycin resistance in *E. casseliflavus* strain SSK.

Modification in the peptidoglycan production pathway, specifically the substitution of d-Ala-d-Ala for d-Ala-d-Lac or d-Ala-d-Ser, is the main cause of vancomycin resistance in enterococci.^12,13,76^ Such changes are attributed to genes found on mobile genetic elements and/or chromosomally encoded sections of different Enterococcus species. Enterococcus species harbor nine distinct vancomycin resistance genes: vanA, vanB, vanC, vanD, vanE, vanG, vanL, vanM, and vanN.^5,16−21,77^ Among these, vanC, vanE, vanG, vanL, and vanN confer low-level vancomycin resistance.^17−20,48,58^ Thus, understanding their mechanism is crucial for developing strategies to combat antibiotic resistance. The gene detection studies confirmed the presence of the vanC2 gene in *E. casseliflavus* SSK, corroborating its modest vancomycin resistance profile (MIC = 2–32 μg/mL) alongside retained sensitivity to teicoplanin.^58,78,79^ The antibiotic susceptibility profile of *E. casseliflavus* SSK indicates exclusive resistance to multiple aminoglycosides, co-trimoxazole, and nalidixic acid. However, fluoroquinolones and co-trimoxazole are recommended for empirical treatment of urinary tract infections (UTIs).^80^ The purified fusion protein had a molecular mass of approximately 39 kDa, aligning with findings by Park et al.^58^ and the predicted 38.35 kDa for EcfDdls by the ExPASy ProtParam server based on the vanC2 sequence, including the 0.8 kDa for the 6× His tag. This exploration delved into the mechanistic insights behind vancomycin resistance, focusing on EcfDdls, responsible for catalyzing the dimerization of d-Ala and d-Ser residues.^58,81^

Peptidoglycan biosynthesis crucially depends on the activities of Ddl and its mutant forms, particularly vanA, vanB, and vanC. The enzymes associated with d-Ala-d-Ala ligase demonstrate specific preferences for substrates, typically generating the d-Ala-d-Ala dipeptide. In vancomycin-resistant Gram-positive bacteria, related enzymes produce the d-Ala-d-Lac or d-Ala-d-Ser dipeptide.^14,82,83^ Determining the specificity of EcfDdls for substrates d-Ala, d-Ser, and d-Lac at subsites 1 and 2 is crucial for comprehending the enzyme’s function and developing potential drugs to alleviate vancomycin resistance. EcfDdls has been demonstrated to synthesize the d-Ala-d-Ala dipeptide at an optimal pH of 8. Conversely, the vanC2 enzyme from *E. casseliflavus* ATCC 25788 was reported not to exhibit significant d-Ala-d-Ala ligase activity at pH 7.5.^58^ Additionally, EcfDdls cannot synthesize the d-Ala-d-Lac dipeptide, as evidenced by the absence of released inorganic phosphate in reactions involving d-Ala and d-Lac substrates.^58^ The *K*_m1_ for d-Ala at subsite 1 was determined to be 1.12 ± 0.1 mM, closely aligned with previously reported values of 1.6 and 1.8 mM.^14,58^ The *K*_m2_ for d-Ser was 0.8 ± 0.1 mM, indicating higher specificity for d-Ser at the second subsite. Thus, enzyme characterization studies unveiled EcfDdls’ substrate specificity for d-Ala and d-Ser, with kinetic analysis affirming its affinity at subsites 1 and 2 for these substrates.^14,58^ The half-minimal inhibitory concentration (IC_50_) of a medicine is crucial to ensure its safe and effective use. The findings highlighted the inhibitory potential of a virtually screened oxadiazole derivative (CID 45805715) from the earlier study,^53^ exhibiting promising efficacy (IC_50_ = 76.7 ± 1.01 μM) against the purified vanC2 EcfDdls enzyme, comparable to the established inhibitor DCS (IC_50_ = 313 ± 1.6 μM). This value aligns with the IC_50_ value for *E. coli* DdlB (320 μM)^84^ and *Mycobacterium tuberculosis*d-Ala-d-Ala ligase MtDDL (370 μM).^85^ However, Park et al. reported an 800 μM IC_50_ value of DCS against vanC2.^58^

Beyond *in vitro* enzyme inhibition, assessing a drug molecule’s ability to impede bacterial growth is paramount.^34^ Most drug molecules screened against Ddl fail to penetrate the bacterial cell wall.^34^ Remarkably, the inhibitor CID 45805715 demonstrated an MIC value of 128 μg/mL against *E. casseliflavus* strain SSK, positioning it as a potential contender in combating vancomycin-resistant bacterial infections. DCS, a substrate analogue (d-Ala analogue) Ddl inhibitor, has an MIC value of 32 μg/mL and is the only FDA-approved drug as a second-line therapeutic agent against multidrug-resistant *Mycobacterium tuberculosis* (MDR). However, neurotoxic side effects limit its use.^34^ Thus, these findings advocate for further exploration of 1-[(5-methyl-1,2-oxazol-3-yl)methyl]-4-{[3-(propan-2-yl)-1,2,4-oxadiazol-5-yl]methyl}piperazine (CID 45805715) as a promising therapeutic agent to mitigate the threat posed by vancomycin-resistant bacteria.

## 5 Conclusions

In summary, the present study highlights the critical role of d-alanine-d-alanine ligases (Ddl) and their isozymes in the development of vancomycin resistance among Gram-positive bacteria, particularly Enterococcus species. The identification and purification of the vanC2 type of d-alanine-d-serine ligase (EcfDdls) from the vancomycin-resistant *Enterococcus casseliflavus* strain SSK, along with the comprehensive analysis of substrate specificity and enzyme kinetics, have elucidated the enzyme’s mechanism of action. Investigation of the oxadiazole derivative (CID 45805715) demonstrated its full inhibitory effect on EcfDdls and its antimicrobial activity against the vancomycin-resistant strain. These findings underscore the potential of this compound as a promising candidate for the development of new antibacterial therapies aimed at mitigating vancomycin resistance in enterococci. Ultimately, this research provides valuable insights into the inhibition of vanC2 EcfDdls and offers a foundation for future studies aimed at combating nosocomial infections caused by vancomycin-resistant bacteria, thereby contributing to improved treatment outcomes for immunocompromised patients.

## Supporting Information Available

Complete set of experimental data: cloning plan, map of the vector, colorimetric assay of Ddl, microscopic examination of isolate S6, confirmation of the positive clone, plot of standard inorganic phosphate, and antibiotic susceptibility of isolate S6 (PDF)
