# Supplementary material for: Overexpression, Purification, and Biochemical Characterization of the vanC2 d-Ala-d-Ser Ligase from Enterococcus casseliflavus SSK and Its Inhibition by an Oxadiazole Derivative
Source: ACS Omega. 2025 Apr 3;10(14):14390–402. doi: 10.1021/acsomega.5c00599 (PMC12004154; doi:10.1021/acsomega.5c00599)
Supplement: Supplementary file 1 — ao5c00599_si_001.pdf [file ao5c00599_si_001.pdf]

Overexpression, purification, biochemical characterization of VanC2 D-Ala-D-Ser ligase from *Enterococcus casseliflavus* SSK and its inhibition by oxadiazole derivative

Sneha B. Paymal[a,b], Sagar S. Barale[c], Shirishkumar V. Supanekar*[b], Kailas D. Sonawane*[d], Kiran D. Pawar*[e].

[a]Department of Microbiology, Shivaji University, Vidyanagar, Kolhapur 416004, Maharashtra, India

[b]Rayat Institute of Research and Development (RIRD), Satara 415001, Maharashtra, India

[c]Department of Microbiology, School of Life Sciences, Central University of Rajasthan, Ajmer 305817, Rajasthan, India.

[d]Department of Biochemistry, Shivaji University, Vidyanagar, Kolhapur 416004, Maharashtra, India.

[e]School of Nanoscience and Biotechnology, Shivaji University, Vidyanagar, Kolhapur 416004, Maharashtra, India.

Running Head: Ddl inhibition to control vancomycin resistance.

*Corresponding author:

Dr. Kiran D. Pawar

Assistant Professor,
School of Nanoscience and Biotechnology,
Shivaji University, Vidyanagar, Kolhapur (MS) 416004, India.
Email: kdp.snst@unishivaji.ac.in; pawarkiran1912@gmail.com
Phone: +91-7972147331

Prof. (Dr.) Kailas D. Sonawane

Professor and Head,
Department of Biochemistry,
Shivaji University, Vidyanagar, Kolhapur (MS) 416004, India.
Email: kds_biochem@unishivaji.ac.in
Phone: +91-9881320719

Dr. Shirishkumar V. Supanekar

Rayat Institute of Research and Development (RIRD),
Satara (MS) 415001, Maharashtra, India
Email: svsupanekar@rediffmail.com
Phone: +91-9422592993

Supplementary material

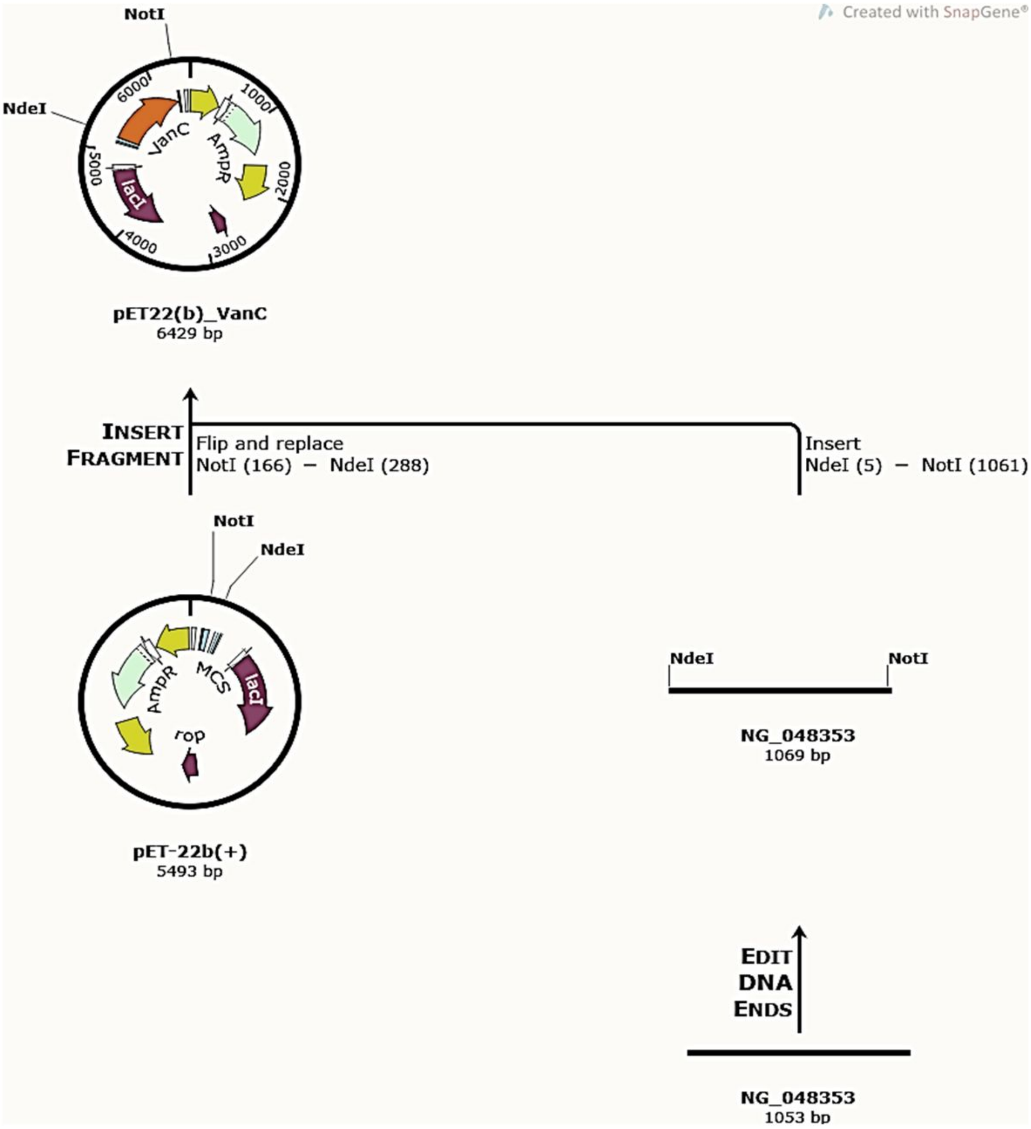

Fig. S1 The flowchart of recombinant pET22b-vanC2 plasmid construction.

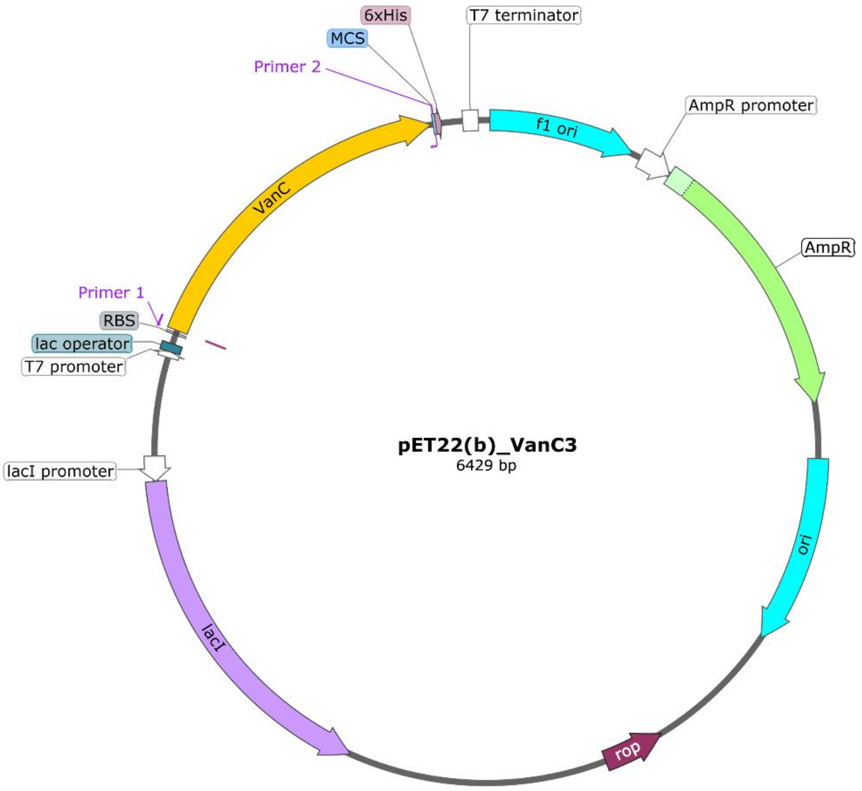

Fig. S2 Complete map of pET22b-vanC2 plasmid. The *vanC2* gene was cloned using NdeI and NotI retriction sites of pET22b(+) to incorporate 6xHis tag during expression. The complete recombined map was created with SnapGene.

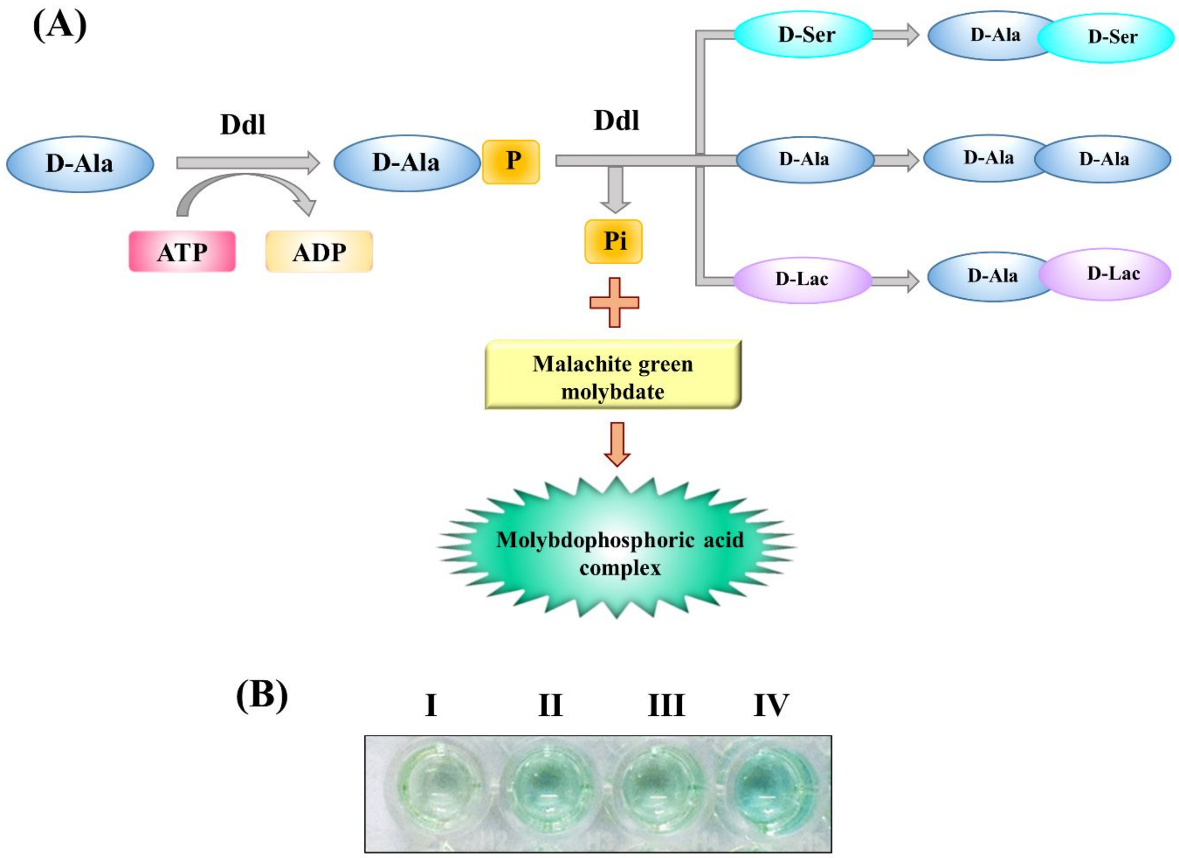

Fig. S3 The colorimetric assay of Ddl. (A) The scheme of the colorimetric assay. Ddl catalyses dimerization of D-amino acids by coupling of ATP hydrolysis, and the final product Pi molecules were detected by the malachite green reagent. (B) The results of the colorimetric assay. Well (I) Blank, (II) Enzyme control, (III) Substrate control, and (IV) Test

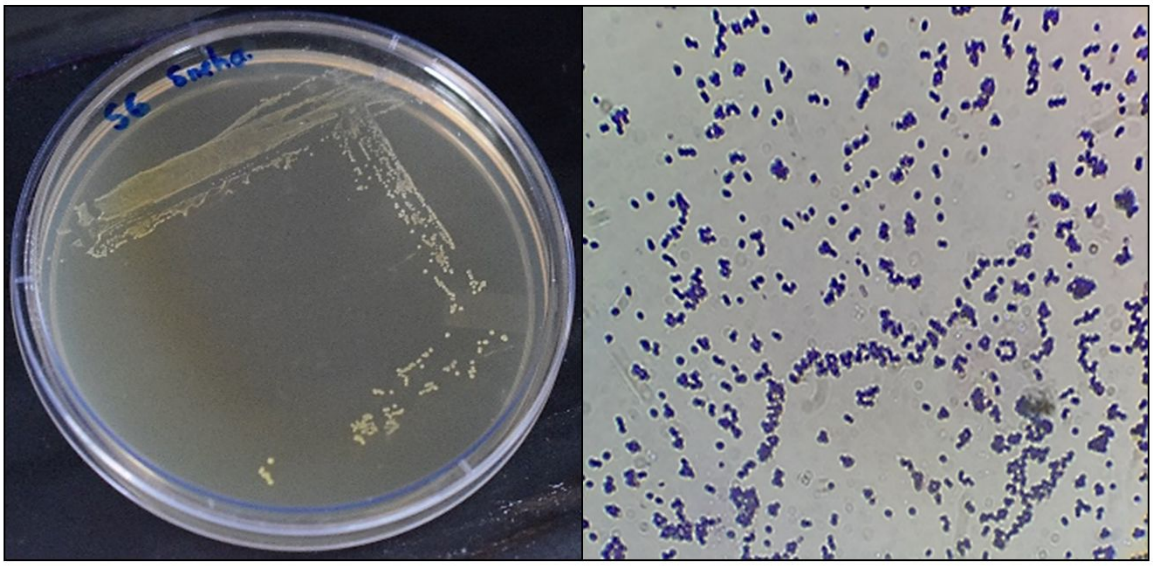

(A) **(B)**

Fig. S4 (A) Colony morphology of vancomycin resistant isolate S6 on BHI-Vancomycin Agar after 24 h incubation at 37 °C (B) Gram staining property of isolate S6

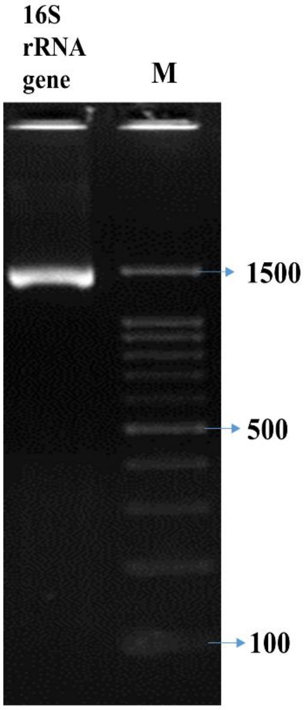

Fig. S5 Analysis of 16S rRNA gene by PCR; M- Molecular size marker (BioLit ProxiB 100bp DNA ladder, SRL, India).

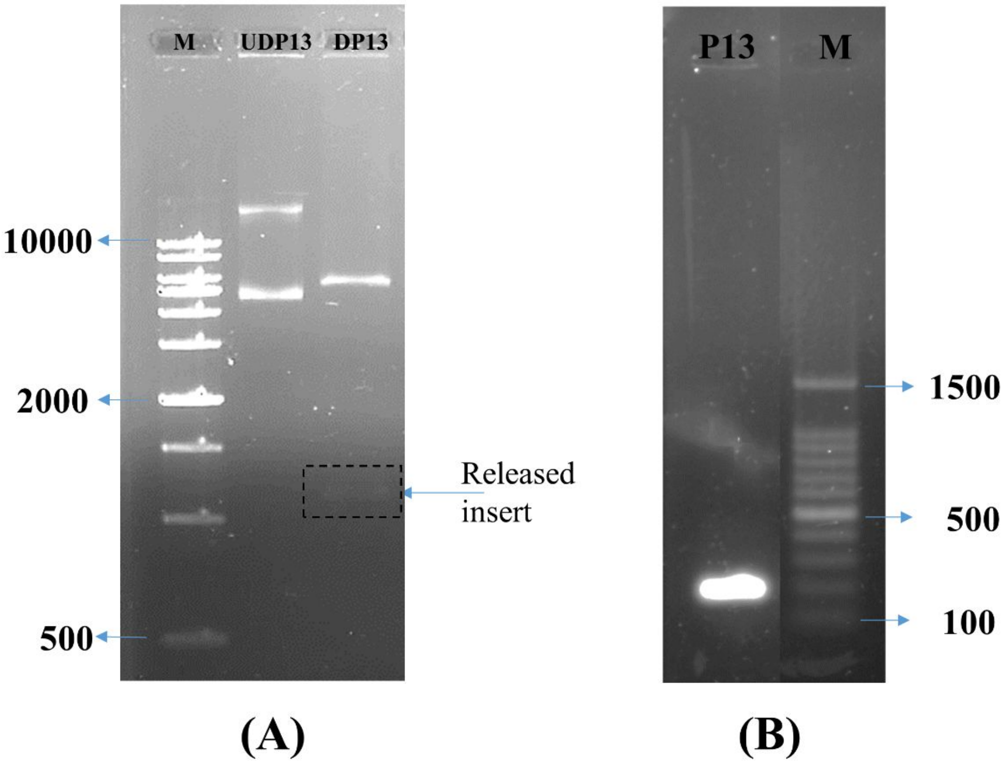

Fig. S6 Confirmation of positive clone by (A) Restriction analysis - Restriction digestion of pET22b-vanC2 construct by Nde1 and Not1 enzymes; M- Molecular size marker (BioLit ProxiB 1kb DNA ladder, SRL, India.), UDP13-undigested selected clone, DP13-restriction digested selected clone and (B) PCR-based *VanC2* gene detection;P13- PCR product of selected clone, M- Molecular size marker (BioLit ProxiB 100bp DNA ladder, SRL, India.).

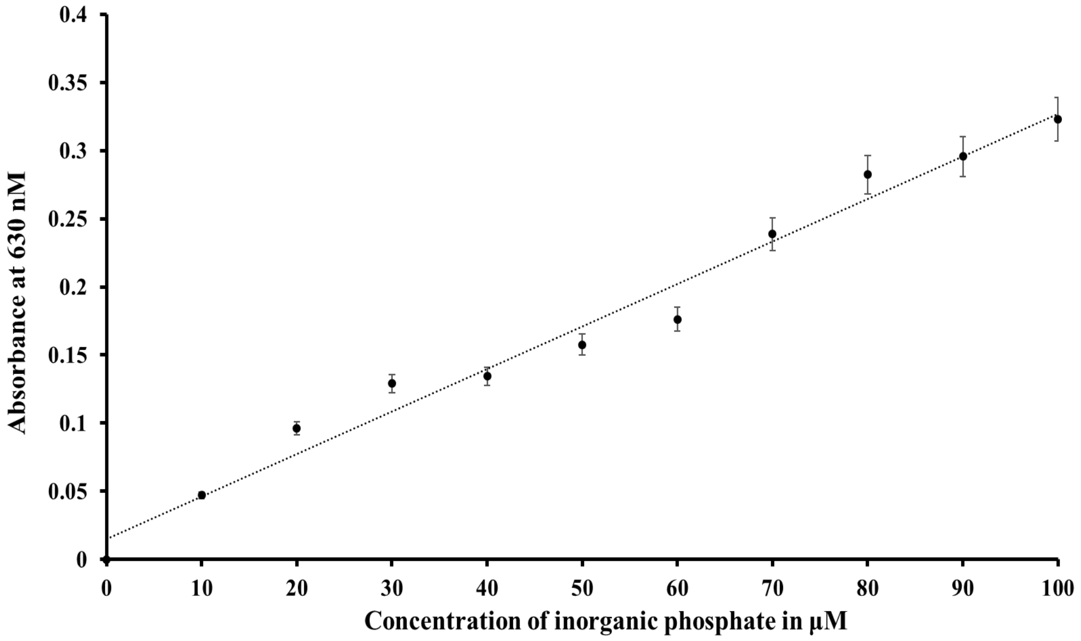

Fig. S7 Plot representing standard inorganic phosphate (concentration in µM).

Table S1 Antibiotic susceptibility of isolate S6 against standard antibiotics

**Antibiotics (µg/disk)**	**Zone of inhibition (mm)**
Teicoplanin (TEI) (30)	25 ± 0.4
Clarithromycin (CLR) (15)	24.33 ± 0.2
Gentamicin (GEN) (10)	20 ± 0.4
Streptomycin (S) (10)	0 ± 0.0
Nitrofurantoin (NIT) (300)	18 ± 0.4
Co-Trimoxazole (COT) (25)	0 ± 0.0
Amikacin (AK) (30)	7.33 ± 0.2
Tobramycin (TOB) (10)	8.17 ± 0.2
Oxytetracycline (O) (30)	22 ± 0.0
Furazolidone (FR) (50)	23 ± 0.0
Netillin (NET) (30)	13 ± 0.4
Kanamycin (K) (30)	13.17 ± 0.2
Nalidixic Acid (NA) (30)	0 ± 0.0
